# Uterine luminal-derived extracellular vesicles: potential nanomaterials to improve embryo implantation

**DOI:** 10.1186/s12951-023-01834-1

**Published:** 2023-03-07

**Authors:** Linjun Hong, Xupeng Zang, Qun Hu, Yanjuan He, Zhiqian Xu, Yanshe Xie, Ting Gu, Huaqiang Yang, Jie Yang, Junsong Shi, Enqin Zheng, Sixiu Huang, Zheng Xu, Dewu Liu, Gengyuan Cai, Zicong Li, Zhenfang Wu

**Affiliations:** 1 National Engineering Research Center for Breeding Swine Industry, College of Animal Science, South China Agricultural University, Guangzhou, 510642 People’s Republic of China; 2 Guangdong Provincial Key Laboratory of Agro-Animal Genomics and Molecular Breeding, College of Animal Science, South China Agricultural University, Guangzhou, 510642 People’s Republic of China; 3 Guangdong Laboratory for Lingnan Modern Agriculture, Guangzhou, 510642 People’s Republic of China; 4 Yunfu Subcenter of Guangdong Laboratory for Lingnan Modern Agriculture, Yunfu, 527300 People’s Republic of China; 5 State Key Laboratory of Livestock and Poultry Breeding, Guangzhou, 510642 People’s Republic of China

**Keywords:** Uterine luminal fluid, Extracellular vesicles, Nanomedicine materials, Implantation, MEP1B

## Abstract

Most pregnancy losses worldwide are caused by implantation failure for which there is a lack of effective therapeutics. Extracellular vesicles are considered potential endogenous nanomedicines because of their unique biological functions. However, the limited supply of ULF-EVs prevents their development and application in infertility diseases such as implantation failure. In this study, pigs were used as a human biomedical model, and ULF-EVs were isolated from the uterine luminal. We comprehensively characterized the proteins enriched in ULF-EVs and revealed their biological functions in promoting embryo implantation. By exogenously supplying ULF-EVs, we demonstrated that ULF-EVs improve embryo implantation, suggesting that ULF-EVs are a potential nanomaterial to treat implantation failure. Furthermore, we identified that MEP1B is important in improving embryo implantation by promoting trophoblast cell proliferation and migration. These results indicated that ULF-EVs can be a potential nanomaterial to improve embryo implantation.

**Supplementary Information:**

The online version contains supplementary material available at 10.1186/s12951-023-01834-1.

## Introduction

Embryo implantation is a critical process to ensure a successful pregnancy to term, which requires the acquisition of receptive endometrium and timely development of an embryo [1]. There are numerous reasons for miscarriage, including environmental factors, obesity, and uterine pathologies such as polyps and myomas, and implantation failure is one of the main causes of pregnancy loss [2, 3]. According to statistics, approximately 78% of pregnancy loss results from implantation failure [4]. Although some progress has been made in studying implantation failure [5], there are still great challenges in clarifying this issue, and there is a lack of effective therapeutics. Consequently, there is an urgent need to identify new options for treating implantation failure.

EVs, with diameters of approximately 40–150 nm, are derived from endosomes or plasma membranes that can participate in various biological processes and facilitate intercellular communication via exchanging various cargoes such as proteins, nucleic acids, and lipids [6–8]. These nano-scale vesicles are considered ideal endogenous materials for nanomedicine because of their natural biological functions [9]. Previous studies have demonstrated that EVs derived from uterine luminal fluid (ULF) could stimulate maternal–fetal crosstalk, thereby enhancing embryonic development [10]. Notably, endometrium-derived EVs can be ingested by embryos and change their protein composition, thereby increasing their implantation and invasion potential [11], indicating that ULF-EVs may be potential materials for improving embryo implantation and treating implantation failure.

However, because the uterine luminal fluid of normal pregnant women is almost difficult to obtain, the limited supply of human ULF severely prevents the production of large amounts of ULF-EVs. Pigs are often used as biomedical models for human studies because of their similarities in anatomical size, structure, physiology, and genome [12]. The wide availability, long embryo implantation period, and established protocols for manipulating the reproductive cycle of pigs make their uterine luminal fluid a potential source of ULF-EVs [13]. However, proteins that directly participate in biological processes have not been clearly characterized in pig ULF-EVs, which seriously hinders the development of these ideal nanomaterials in treating human infertility diseases.

Here, we isolated EVs from porcine ULF during the implantation stages (including day 9 of pregnancy for the pre-implantation stage, day 12 of pregnancy for the maternal–fetal pregnancy recognition, and day 15 of pregnancy for the embryo attachment stage [14]) using differential centrifugation and density gradient ultracentrifugation and comprehensively characterized protein abundance differences in these EVs. We then investigated whether these isolated ULF-EVs caused changes in implantation-related biological functions. Moreover, we identified MEP1B protein enriched in EVs and explored the potential molecular mechanisms for improving embryo implantation.

## Materials and methods

### Animals and sample collection

Twelve healthy and disease-free Yorkshire sows (parity 2) were purchased from Wen's Foodstuffs Group Co., Ltd. (Yunfu, China). All sows were randomly divided into two groups: cyclic (n = 3) and pregnant (n = 9). All animals were examined for estrus twice a day, and those in the pregnant group were artificially inseminated with a standard dose of single Yorkshire semen after estrus. By contrast, those in the cyclic group were artificially inseminated with dead semen from the same boar. On day 9 of the estrous cycle (9C, n = 3) and days 9, 12, and 15 of pregnancy (9P, 12P, and 15P, n = 3 sows/day of pregnancy), sows were slaughtered at a nearby slaughterhouse. The uterus was extracted swiftly and transported to the laboratory in an icebox. Approximately 1 cm^2^ of uterine section samples were collected from each uterine horn on the antimesometrial side of uterus. They were quickly fixed in 10% neutral-buffered formalin for 24 h for paraffin embedding (FFPE), hematoxylin–eosin (H&E) staining, periodic acid-Schiff (PAS) staining, and immunohistochemistry (IHC). Each uterine horn was flushed with 200 mL sterile phosphate-buffered saline (PBS, pH = 7.2), and pregnancy was established by the appearance of normal spherical (day 9 of pregnancy) or filamentous embryos (days 12 and 15 of pregnancy) (Additional file 1: Fig. S1). The media were centrifuged at 4000×*g* for 5 min to remove cell debris. For subsequent tests, the embryos or uterine luminal fluid samples were quickly frozen in liquid nitrogen and preserved at − 80 °C.

### H&E staining

Embedded uterine samples were sliced into 5 µm thick sections. The sections were subjected to H&E staining for histological inspection. Nikon 80i microscope was used to observe and photograph the images (Nikon, Tokyo, Japan).

### PAS staining

Uterine sections were deparaffinized and submerged for 10 min at room temperature in a periodic acid solution. After rinsing the sections with free-flowing water for a few minutes, they were incubated in Schiff reagent for 10 min in the dark. After rinsing with running water for 5 min, sections were counterstained with hematoxylin solution for 90 s. After staining, samples were rinsed. After air-drying the slides, neutral gum was added dropwise to mount them. Sections were viewed and photographed using an Olympus BX-53 microscope equipped with a DP26 digital camera.

### Isolation of ULF-EVs

ULF-EVs were extracted from ULF samples by employing OptiPrep™ density gradient ultracentrifugation (ODG UC) [15] as described previously. Briefly, the samples were centrifuged at 10,000×*g* for 30 min to remove macroparticles and apoptotic bodies and then ultracentrifuged at 100,000×*g* for 2 h using an SW41T rotor (Beckman Coulter Instruments, Fullerton, CA, USA) to precipitate ULF-EVs twice. The final pellet was resuspended in 100 µL phosphate-buffered saline (PBS) (Gibco, USA). All centrifugation processes were conducted at 4 °C.

Discontinuous iodixanol gradients of 5%, 10%, 20%, and 40% iodixanol were formed in polyallomer tubes by diluting OptiPrep™ (60% (w/v) aqueous iodixanol solutions) with an appropriate amount of 0.25 M sucrose and 10 M tris as previously described (Beckman Coulter Instruments, Fullerton, CA, USA) [16]. The pellet was then covered and centrifuged for 18 h at 100,000×*g* at 4 °C (Beckman Coulter Instruments, Fullerton, CA, USA). The 12 stratification fractions were collected and diluted in PBS to eliminate any remaining OptiPrep™, and each fraction was ultra-centrifuged for 3 h at 100,000×*g* and 4 °C. The pellet was re-suspended and stored at − 80 °C for subsequent tests, and ULF-EVs were primarily in the layer between fractions 7 ~ 11.

### Transmission electron microscope (TEM)

A suspension of 10 µL of ULF-EVs was evenly spread over a 200-mesh copper grid and allowed to stand for 1 min. Subsequently, 10 µL of 2% aqueous uranyl acetate was added, and the mixture was incubated for 2 min. Grids were air-dried at ambient temperature, and respective sections were viewed and photographed at an acceleration voltage of 80 kV using a transmission electron microscope (FEI Talos F200S).

### Nanoparticle tracking analysis (NTA)

ULF-EV suspension was diluted 4000 times with PBS, and then 2 mL of the diluted ULF-EV suspension was loaded into the chamber. The size distribution of ULF-EVs was determined using ZetaView PMX 110 (Particle Metrix, Germany) and the corresponding ZetaView 8.04.02 software for nanoparticle tracking analysis.

### Protein extraction, enzymatic hydrolysis, and RPLC-MS/MS analysis

Samples were transferred into low-protein-binding tubes (1.5 mL). Samples were subsequently lysed in 300 µL SDS lysis buffer (Beyotime Biotechnology, Shanghai, China) containing 1 mM PMSF (Amresco, USA), a protease inhibitor. Sonication on ice was used to further lyse the samples, with parameters set at 1 s/1 s intervals, for a time of 3 min, and power of 80 W. After sonication, samples were centrifuged for 10 min at 12,000×*g* to remove insoluble particles. To further exclude precipitation, centrifugation was performed once more. The protein content was measured using BCA test (Beyotime Biotechnology, Shanghai, China). Each protein sample weighed 10 µg and was separated on a 12% SDS-PAGE gel. CBB was used to dye the separating gel according to Candiano’s protocol [17]. The stained gel was scanned at 300 dpi using an ImageScanner (GE Healthcare, USA).

The enzymatic hydrolysis of proteins was accomplished using FASP protocol [18]. Each protein sample (100 µg) was added to 120 µL of reducing buffer (10 mM DTT, 8 M Urea, 100 mM TEAB, pH = 8.0) (Sangon Biotech, Shanghai, China) and transferred into a 10 K ultrafiltration tube. The solution was incubated at 60 °C for 1 h before IAA addition to achieve a final concentration of 50 mM in the dark for 40 min at room temperature. The solution was centrifuged for 20 min at 4 °C at 12,000 rpm to obtain a precipitate. After adding 100 µL of 300 mM TEAB buffer to the solution, it was centrifuged twice at 12,000 rpm for 20 min. Following washing, the filter unit was placed in a fresh collection tube. Each tube was then filled with 100 µL of 300 mM TEAB buffer, followed by adding 2 µL of sequencing-grade trypsin (1 µg/µL). The solution was then incubated for 12 h at 37 °C for enzymatic hydrolysis. The digested peptides were collected and centrifuged for 20 min at 12,000 rpm. The mixture was centrifuged after adding 50 µL of 200 mM TEAB. The precipitate was lyophilized once it was collected.

RP separation was performed on an Agilent 1100 HPLC System (Agilent, USA) using an Agilent Zorbax Extend RP column (5 µm × 150 mm, 2.1 mm). The mobile phase consisted of a 2% acetonitrile aqueous solution (A) and 90% acetonitrile aqueous solution (B) for RP gradient. The solvent gradient was set as follows: 0–8 min, 98% A; 8.00–8.01 min, 98–95% A; 8.01–38 min, 95–75% A; 38–50 min, 75–60% A; 50–50.01 min, 60–10% A; 50.01–60 min, 10% A; 60–60.01 min, 10–98% A; 60.01–65 min, 98% A. Peptides were separated at 300 µL/min flow rate and measured at 210 and 280 nm. Dried samples were collected from 8 to 50 min, while the elution buffer was collected every minute and numbered from 1 to 15. The peptides were separated and lyophilized for subsequent MS detection.

Samples were loaded and separated using a C18 column (3 cm × 100 µm, 3 μm, 150 Å) on a TripleTOF 6600 system (SCIEX, USA). The flow rate was set at 300 nl/min, and the linear gradient was set at 50 min (0–0.1 min, 4–6% B; 0.1–32 min, 6–25%B; 32–42 min, 25–38% B; 42–42.1 min, 38–90% B; 42.1–47 min, 90% B; 47–47.1, 90–4% B; 47.1–50 min, 4% B; mobile phase A was 2% ACN/0.1% FA in water and B was 95% ACN/0.1% FA in water). The data were collected with a 2.4 kV ion spray voltage, curtain gas with a pressure of 40 PSI, a nebulizer gas pressure of 12 PSI, and 150 °C interface heater temperature. The mass spectrometer was operated in the information-dependent acquisition mode (IDA, Information Dependent Analysis) with a full MS scan range of 350–1500 m/z, and the scan time was 250 ms. The 42 most intense peaks in MS were fragmented in MS/MS spectra scan range of 100–1500 m/z, and the scan time was 50 ms. The collision energy setting was used for all precursor ion collision-induced dissociation (CID). Dynamic exclusion was set at 14.0 s.

### Proteomic data analysis

Three biological replicates were created to boost the credibility of analysis. MaxQuant (v.1.3.0.5) was used to perform a database search of the raw data. A search of the Uniprot-proteome_UP000008227-Susscrofa database was conducted using trypsin digestion specificity, and a maximum of two missed cleavages were permitted. Cysteine carbamidomethylation was set as a fixed parameter, whereas methionine oxidation, tyrosine, serine, threonine phosphorylation, and protein N-terminal acetylation were specified as variable modifications. The mass deviation of fragments was adjusted to 20 ppm. Additionally, the minimum length of the peptide was set to seven amino acids. The false discovery rate (FDR) was limited to 1% at the peptide level, and proteins contained at least one unique peptide. BUSCA database (http://busca.biocomp.unibo.it) was used to annotate proteins to investigate the proteome’s subcellular localization [19]. Protein abundance patterns in extracellular vesicles of porcine ULF were assessed throughout four different periods using Mfuzz R software to cluster these proteins [20]. Protein clusters were selected to analyze up-and-down-regulation during development. To investigate the functions of the two clusters, GO enrichment and KEGG pathway analyses were performed using clusterProfiler R package [21]. Furthermore, STRING database (http://string-db.org) was used to analyze the protein–protein interaction network (PPI) of these proteins. Cytoscape displayed PPI network (v.3.7.2) [22].

### Western blotting (WB)

Towbin transfer buffer was used to transfer 10 µg of EV protein separated by SDS-PAGE to a PVDF membrane (Merck Millipore, Germany) at 110 V for 70 min. followed by blockade with 5% skimmed milk powder (BD, USA) for 2.5 h. The membranes were incubated with rabbit polyclonal antibodies anti-CD63 (BBI Life Sciences, D160973; 1:1500, China), anti-CD9 (BBI Life Sciences, D164336; 1:1500, China), anti-TSG101 (ZEN BIO, 381,538; 1:1500, China), anti-calnexin (Abcam, ab75801; 1:1500, UK), and monoclonal antibodies anti-MUC4 (Abcam, ab150381; 1:1500, UK), anti-ACP5 (Abcam, ab191406; 1:1500, UK), mouse monoclonal antibodies anti-MEP1B (R&D Systems, MAB28951; 1:1500, USA) overnight at 4 ℃ followed by incubation with secondary antibody for 1.5 h at room temperature. An Ecl Kit (CWBIO, China) was used to visualize immunoreactive bands and was exposed to EC3 Imaging System (UVP).

### Immunohistochemistry

Immunohistochemistry was employed to assess the abundance and distribution of MEP1B in various uterine tissue cells during pregnancy, as described in a recent study [23]. Briefly, 5 µm thick sections were deparaffinized, blocked for 30 min with 5% bovine serum albumin (BSA), then incubated overnight at 4 °C with antibody (R&D Systems, MAB28951, USA). The primary antibody was replaced with purified corresponding immunoglobulin G as a negative control (NC). Secondary antibodies were used to stain the sections and counterstained with hematoxylin. The images were captured using a Nikon 80i microscope (Nikon, Tokyo, Japan), and the average integrated optical density (IOD) was determined using ImagePro Plus 6.0 software (Media Cybernetics, Silver Spring, GA, USA).

### Cell culture and transfection

Cell culture was performed as previously described [24]. Small interfering RNAs (siRNAs) against MEP1B were obtained from GenePharma (Shanghai, China). According to manufacturer’s protocols, full-length MEP1B was amplified and inserted into pcDNA3.1 vector (Invitrogen, Carlsbad, CA, USA) to construct the plasmid, followed by being transiently transfected with Lipofectamine 2000 (Invitrogen, Carlsbad, CA, USA).

### RNA isolation and quantitative real-time PCR (qRT-PCR)

Total RNA was extracted from embryos and cultured cells using the RNeasy Plus Micro Kit (Qiagen, Hilden, Germany) according to the manufacturer’s instructions. The Prime-Script™ RT Master Mix kit (TakaRa, Dalian, China) was used to reverse transcribe these into cDNA. The samples were run on a QuantStudio 7 Flex Real-Time PCR System (Applied Biosystems, Foster City, CA, USA) under standard PCR conditions (an initial step at 94 °C for 2 min, followed by 40 cycles at 94 °C for 30 s, an annealing step at 60 °C for 30 s, extension at 72 °C for 30 s, and a final extension at 72 °C for 5 min). Primers used for qRT-PCR are listed in Additional file 5: Table S1.

### Labeling of ULF-EVs with PKH67

ULF-EVs on day 12 of pregnancy were labeled with PKH67 (Sigma, USA) according to a previously described method [25]. Briefly, ULF-EVs were added to 4 µL PKH67 dye and mixed in buffer for 4 min. Then, the reaction was stopped by adding 2 mL of 0.5% BSA/PBS and ultracentrifuged at 100,000×*g* for 2 h at 4 °C. The labeled ULF-EVs were then suspended in 100 µL PBS and used for subsequent experiments.

### Co-culture experiments of ULF-EVs with pTr2 cells

Labeled ULF-EVs were directly added to the medium containing pTr2 cells for 24 h, and the control group was replaced with PBS. Subsequently, cells were fixed and permeabilized using standard procedures. Phalloidin and DAPI were added for 5 min in the dark to stain the cytoskeleton and nuclei, respectively, and the samples were observed under a confocal laser scanning microscope (LEICA, Germany).

Unlabeled ULF-EVs were added to the medium when pTr2 cells reached approximately 70% of the 6-well plate and continued to culture for 48 h, and the cells were collected for protein extraction. The control group used PBS instead of ULF-EVs.

### Cell counting kit-8 assay

PTr2 cells were seeded onto 96-well plates at a density of approximately 10,000 cells/well. Cells were transfected and incubated for 48 or 72 h at 37 °C with 5% CO_2_. Next, 10 µL of cell counting kit-8 (CCK-8) solution was added, and the culture was continued for 2 h. The absorbance of each well at 450 nm was measured using a microplate reader (Tecan, Switzerland) to determine cell proliferation.

### 5-Ethynyl-2ʹ-deoxyuridine assay

PTr2 cells were seeded at a density of roughly 50,000 cells/well in 24-well plates and cultured overnight. The cells were transfected and incubated for 48 or 72 h at 37 °C with 5% CO_2_. Each well was incubated for 3 h with 5-ethynyl-20-deoxyuridine agent (EdU; BeyoClick, China). The cells were fixed for 15 min in 4% paraformaldehyde, washed with a washing solution, and permeabilized with 0.3% Triton X-100, followed by three PBS washes. A total of 0.3 mL Click was added onto the plate and incubated at room temperature in the dark for 30 min. The nuclear stain DAPI was added, and the number of EdU-stained cells was photographed and visualized using a confocal laser scanning microscope (Leica, Germany).

### Wound healing assay

PTr2 cells were seeded into 6-well plates and cultured at 37 °C with 5% CO_2_. When the cells reached 90% confluence, wounds were created in the monolayer by scratching with a sterile pipette tip and incubating them again. The wound distance was observed and photographed under a microscope after 0 and 24 h of culture.

### Transwell migration assay

Approximately 60,000 pTr2 cells were seeded into the 8 µm upper chamber according to the manufacturer's protocol for Transwell chambers (Corning, New York, NY, USA). The lower chamber was then filled with medium containing 10% FBS and incubated for 24 h. Cells that migrated through the membrane to the lower surface were stained with crystal violet, photographed in a random field, and counted using a light microscope. The assay was repeated thrice.

### Statistical analysis

GraphPad Prism 8.0 (GraphPad Software, San Diego, CA, United States) was used for statistical analysis. Each experiment was performed three times, and the data was expressed as mean ± standard deviation (SD). The Student’s t-test was performed to determine the statistical significance of the two groups. *p* < 0.05 was considered statistically significant, while *p* < 0.01 was considered highly statistically significant.

## Results

### Isolation and characterization of ULF-EVs

**Fig. 1:**
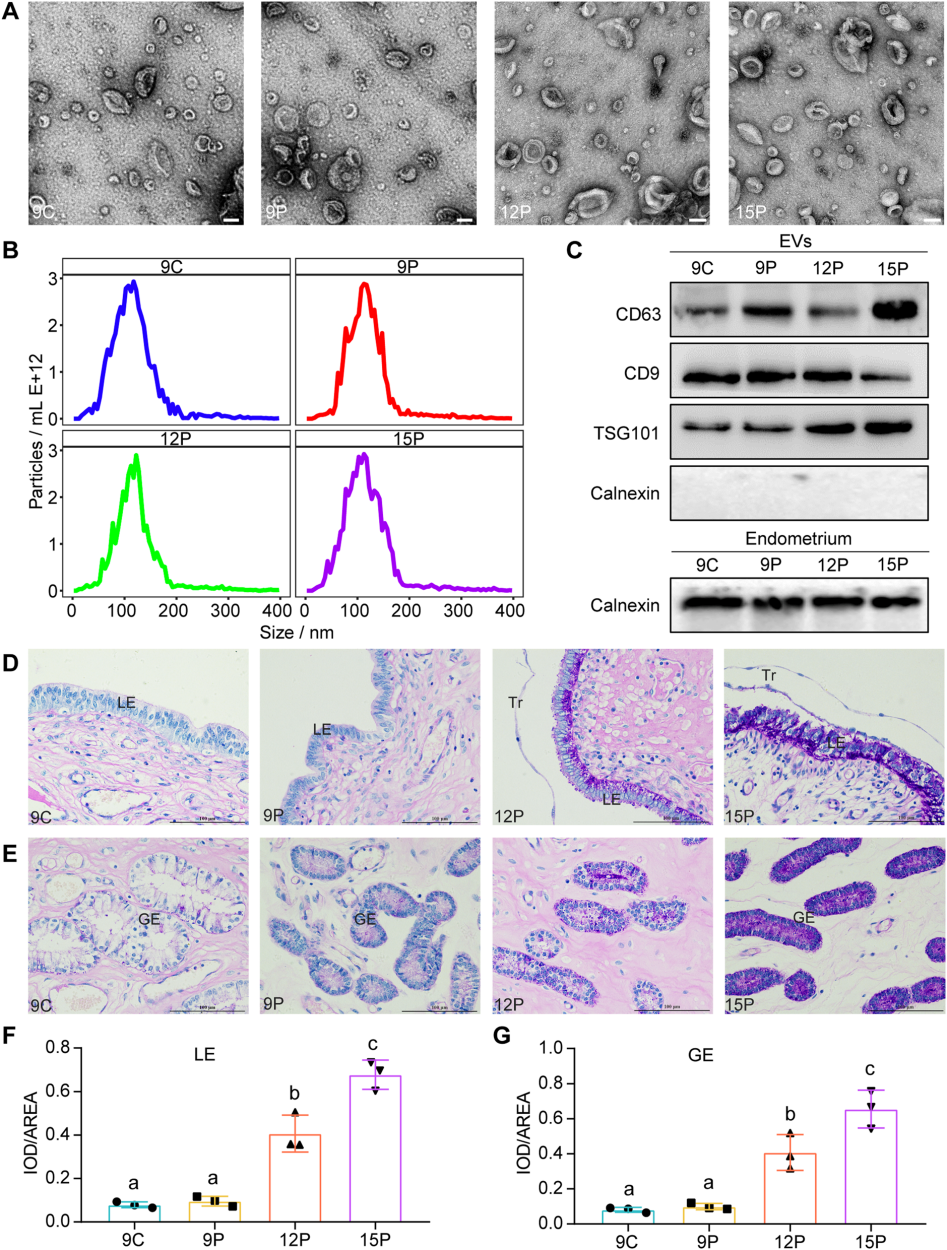
Identification and characterization of ULF-EVs. **A** Transmission electron micrographs of ULF-EVs isolated from days 9, 12 and 15 of pregnancy and day 9 of estrus showed cup-shaped structures with a diameter of approximately 30–150 nm. The scale bar indicates 100 nm. **B** The particle size of ULF-EVs mainly concentrated around 100 nm, which was measured by NTA analysis. **C** Western blotting analysis revealed that ULF-EVs expressed specifical EVs markers CD9, CD63, and TSG101. However, calnexin was extremely enriched in the endometrium compared with corresponding ULF-EV samples. **D**–**G** PAS staining and quantitative analysis of representative uterine sections. As pregnancy progresses, LE or GE could secrete more ULF-EVs. Scale bars = 100 µm. The data are shown as mean ± SD and different lowercase letters correspond to significant differences at the *p* < 0.05 threshold

ULF-EVs were isolated from pig ULF on day 9 of estrus and days 9, 12, and 15 of pregnancy (Additional file 2: Fig. S2). To further characterize the ULF-EVs, we conducted a series of experiments. Figure 1A depicts the morphology of a single ULF-EV, which exhibited a cup-shaped structure with a size range of 30–200 nm. Additionally, NTA analysis revealed that the particle size distribution of ULF-EVs was primarily localized at approximately 100 nm (Fig. 1B). Western blot analysis revealed the presence of CD63, CD9, and TSG101 proteins associated with EV identity but not calnexin (a negative control marker) (Fig. 1C). These results proved that we successfully isolated ULF-EVs.

Since ULF-EVs are secreted by embryo/maternal cells into the uterine luminal, we used PAS staining to determine the secretory activity of uterine tissues. We found that the secretory activity was significantly higher in maternal than in the embryos, and from day 12 of pregnancy, LE and GE had higher secretory activity as the pregnancy proceeded (Fig. 1D–G). In combination with the immunofluorescence staining of EV markers (including CD9 and CD63) of uterine sections from the same stages in our previous study [26], that is, from day 12 of pregnancy, more EVs might be secreted by LE and GE with the progress of pregnancy. The marked increase in ULF-EV secretion suggests that it may play an important role in promoting embryo implantation [11].

### Identification of enriched proteins in ULF-EVs

**Fig. 2:**
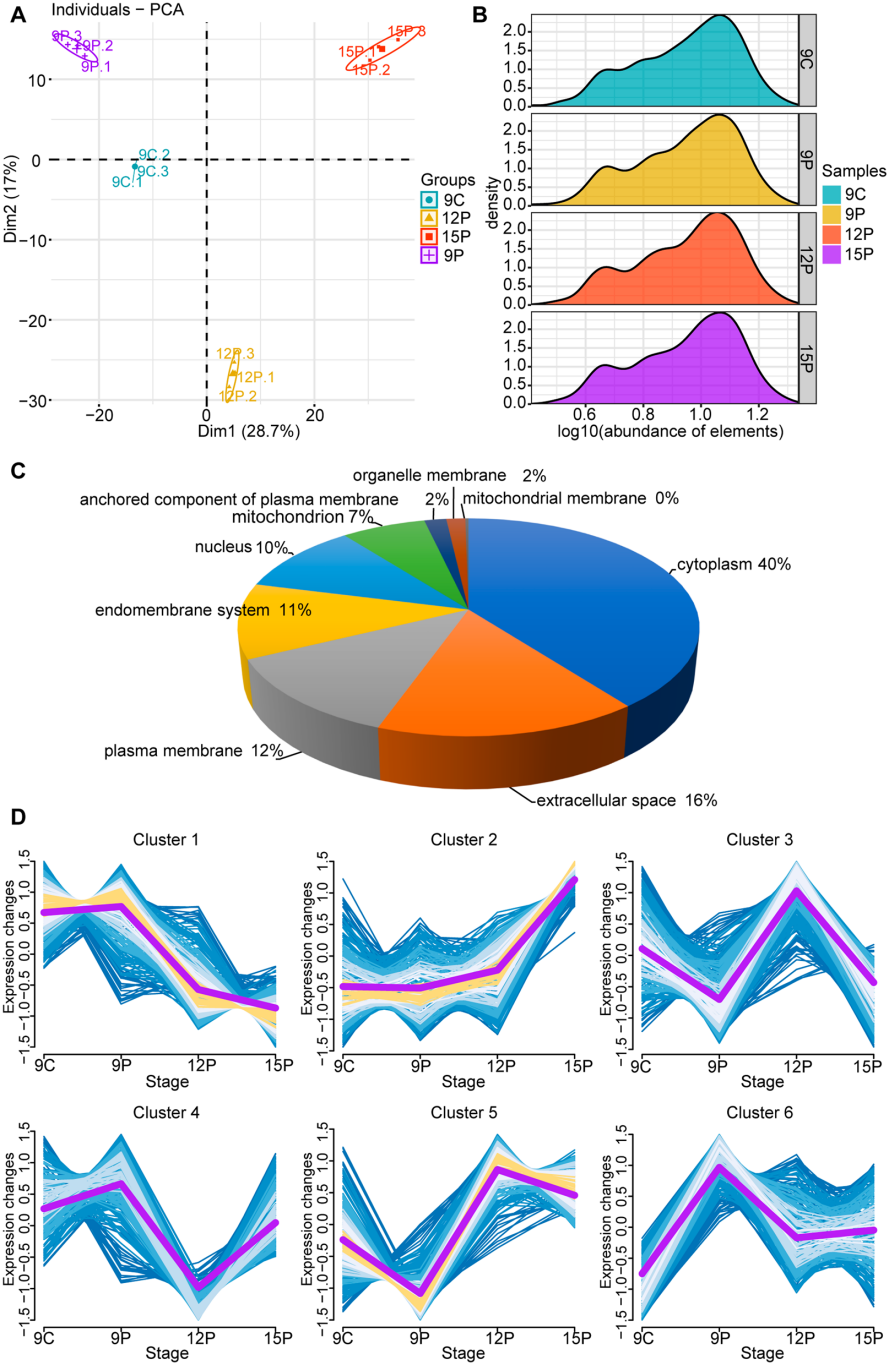
Global identification of proteins in ULF-EVs. **A** Principal component analysis of total proteins in ULF-EVs. **B** Kernel density estimation of the relative abundance of proteins in ULF-EVs in four different periods. **C** Pie chart of intracellular protein locations, which were predicted by BUSCA software. **D** Protein dynamics of ULF-EVs in four different stages. Global proteins were clustered into six soft partitioning clusters, with distinct clusters falling into different biological functions based on abundance patterns. The purple line represents the abundance trend of the proteins in the cluster

To determine the enriched proteins in ULF-EVs on days 9, 12, and 15 of pregnancy and day 9 of estrus, LC–MS/MS analysis was performed. As a result, 1549 proteins were identified (Additional file 6: Table S2). Principal component analysis was conducted to analyze the similarity of proteins within each group (Fig. 2A). High-quality biological repetition is a foundation for further analysis. We found no significant differences in the relative abundance of proteins in ULF-EVs at any of the four-time points (Fig. 2B). Analysis of protein subcellular localization revealed that these proteins were mostly found in the cytoplasm and extracellular space (Fig. 2C). This finding suggests that these proteins can be wrapped and secreted by vesicles to produce ULF-EVs.

To stratify the temporal dynamics of ULF-EVs, we performed unsupervised fuzzy clustering on all enriched proteins from four periods. Six visible protein clusters were found to further investigate the activities of the proteins in ULF-EVs during the four periods, including one up-regulated and one down-regulated with pregnancy development, and four dynamically expressed (Fig. 2D, Additional file 7: Table S3).

### Protein dynamic activity in ULF-EVs

**Fig. 3:**
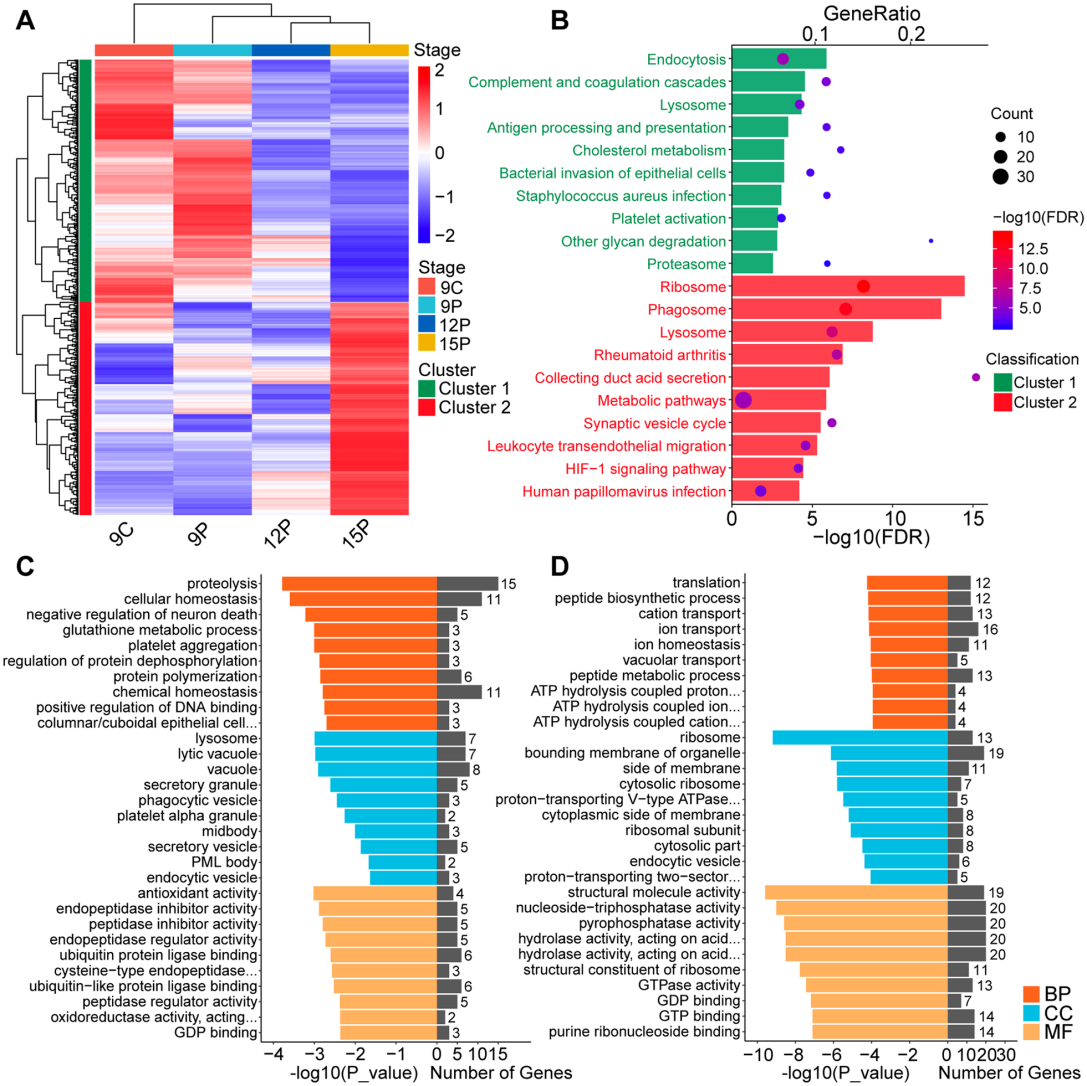
Dynamics of proteins of clusters 1 and 2. **A** Hierarchical clustering analysis of proteins in clusters 1 and 2; cluster 1, the green group, represents proteins whose abundance gradually decreases with pregnancy development, while cluster 2, the red group, represents proteins whose abundance gradually increases. Red in the heatmap indicates an increase in protein abundance, while blue indicates a decrease. **B** KEGG pathway analysis of proteins in clusters 1 and 2. **C** GO enrichment analysis of proteins in cluster 1. **D** GO enrichment analysis of proteins in cluster 2. *BP* biological process, *CC* cellular component, *MF* molecular function

Based on the biological functions of pregnancy that promote embryo implantation, we chose proteins from dynamic clusters 1 and 2 for further functional studies, the abundance level of which increased or decreased with time (Fig. 3A). Cluster 1 (298 proteins) demonstrated a pattern of downregulation as the pregnancy progressed. Proteins in this cluster were mostly involved in protein regulation during development, including proteolysis, glutathione metabolism, protein dephosphorylation regulation, and protein polymerization (Fig. 3C, Additional file 8: Table S4). Cluster 2 (262 proteins) comprised proteins that became more abundant during pregnancy development. Proteins associated with several transport mechanisms, including cation, ion, and vacuolar transport, were enriched, indicating that ULF-EVs play an important role in cellular transport (Fig. 3D, Additional file 8: Table S4). Notably, the functional pathways in clusters 1 and 2 overlapped, including endocytosis, lysosome, ribosome, and phagosome pathways. These ULF-EV proteins also played essential roles in their own synthesis and transport (Fig. 3B, Additional file 8: Table S4). To further investigate the potential relationship between these proteins, STRING database was employed for protein–protein interaction (PPI) analysis (Additional file 3: Fig. S3, Additional file 9: Table S5).

### Proteome analysis of ULF-EVs on pTr2 cells

**Fig. 4:**
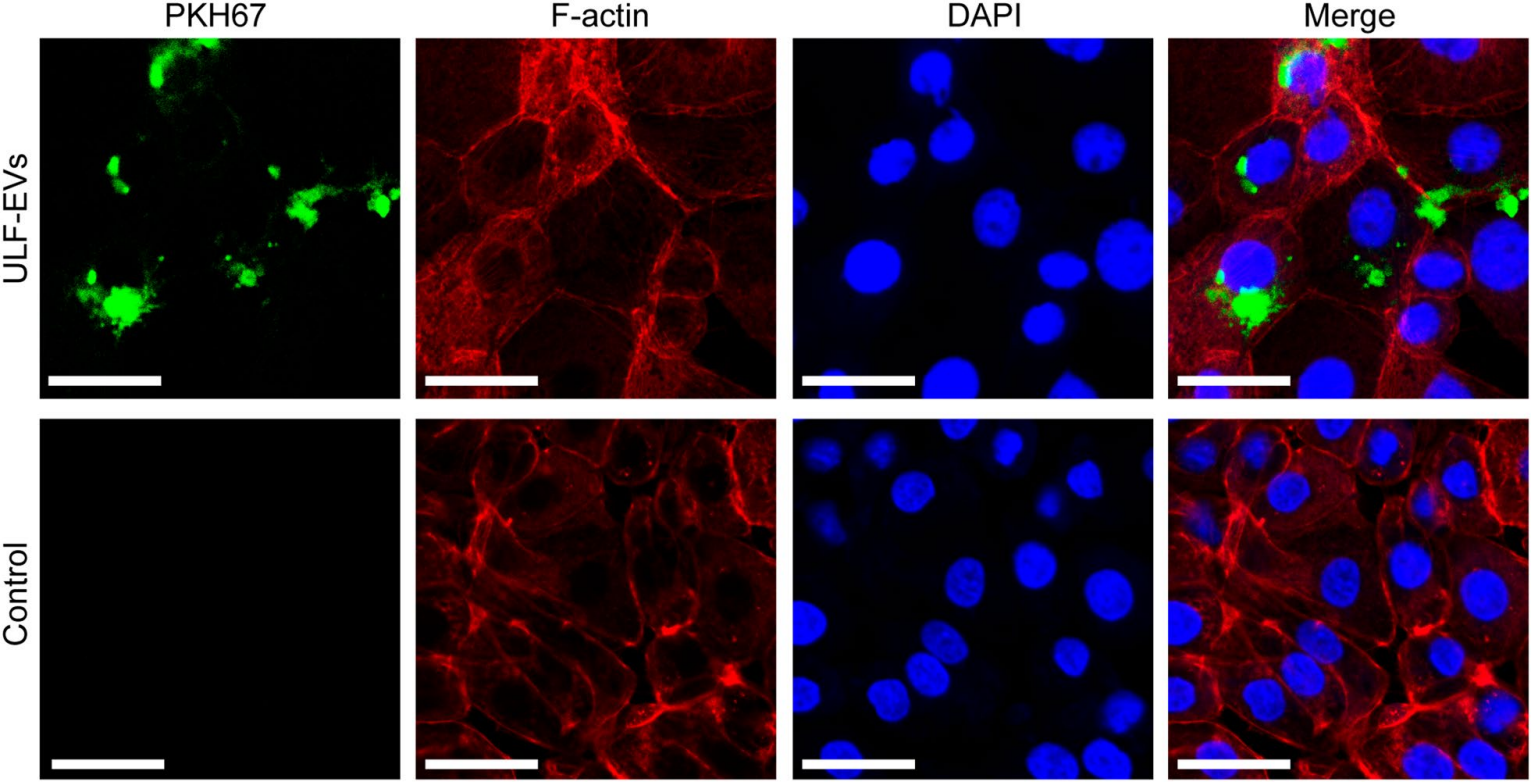
Fluorescence microscopy showing ULF-EVs labeled with PKH67 green fluorescence, cytoskeleton in red, and nuclei in blue (scale bar = 100 μm). For the negative control, an equal volume of PBS was added

The above results proved that ULF-EVs could promote embryo implantation during natural pregnancy. To explore the improvement of embryo implantation by exogenous supplementation of ULF-EVs, considering the secretory activity of ULF-EVs at different stages, we selected ULF-EVs at day 12 of pregnancy to incubate with porcine trophoblast cells (pTr2 cells). Observation under a fluorescence microscope showed that PKH67 fluorescently labeled ULF-EVs could be taken up by pTr2 cells after 24 h and distributed in the cytoplasm. In contrast, fluorescent labeling of PKH67 in the control group was negative, as displayed in Fig. 4.

**Fig. 5:**
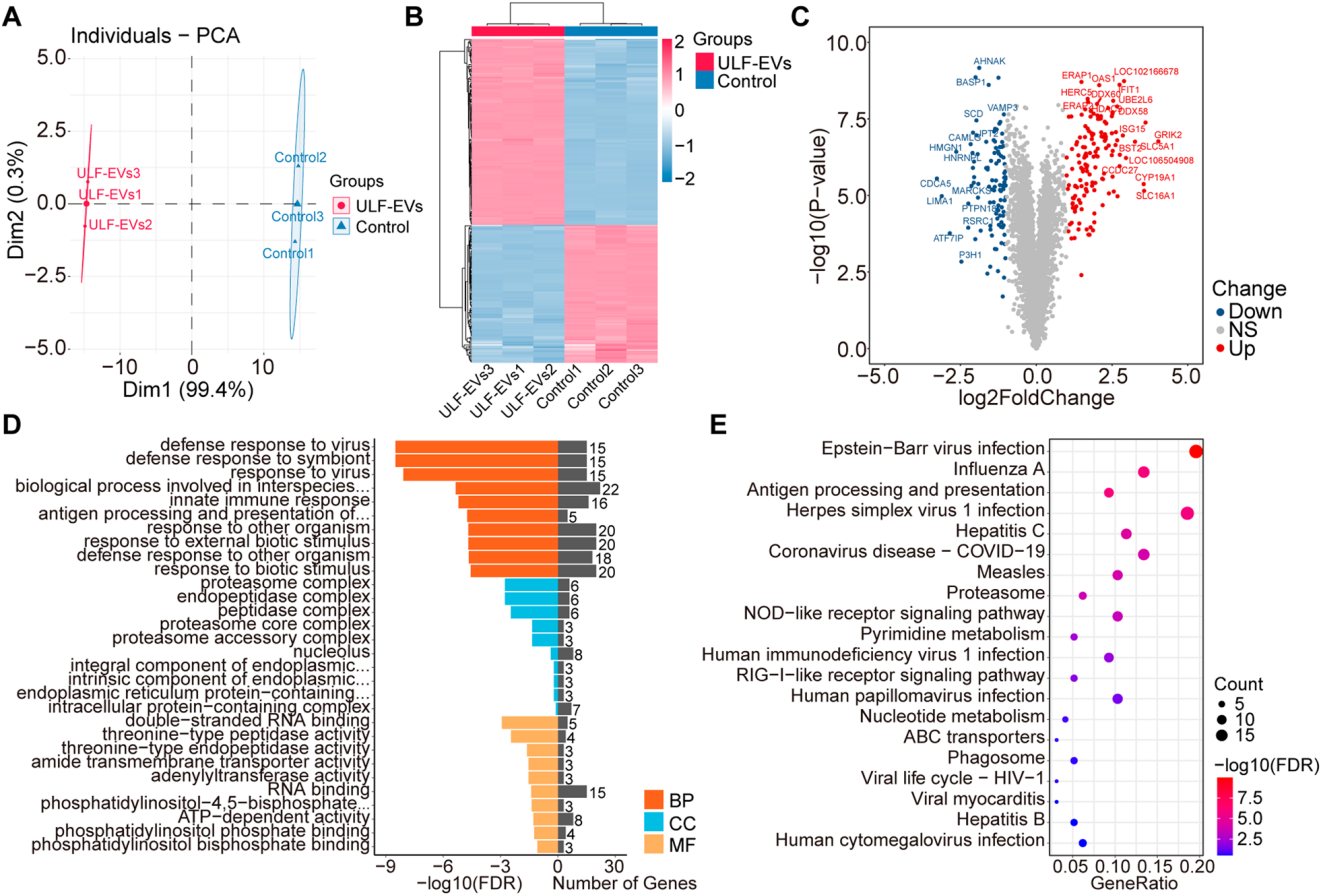
Proteomic analysis of the effects of ULF-EVs on pTr2 cells. **A** Principal component analysis of proteins identified after treatment of pTr2 cells with ULF-EVs. **B** Heatmap of differentially abundant proteins. **C** Volcano plot of differentially abundant proteins. **D** GO enrichment analysis of differentially abundant proteins, showing the top 10 enriched terms for each category. BP, biological process; CC, cellular component; MF, molecular function. **E** KEGG pathway analysis of differentially abundant proteins

We hypothesized that intrauterine supplementation with ULF-EVs could improve embryo implantation. The effect of ULF-EVs on embryo implantation was assessed by comparing the altered protein abundance in pTr2 cells. Principal component analysis demonstrated that the protein abundance in pTr2 cells changed significantly after incubation with ULF-EVs (Fig. 5A). The heatmap depicts the proteins that were significantly up- and down-regulated in ULF-EVs and control groups (Fig. 5B). Compared with the control group, a total of 5542 proteins were identified in the ULF-EVs group, of which 142 were up-regulated and 105 were down-regulated (Fig. 5C). Interestingly, we found that these proteins with significant abundance changes after ULF-EVs treatment were mainly involved in some biological processes and pathways related to immune response, such as innate immune response and antigen processing and presentation (Fig. 5D, E). Previous studies have proved that a common cause of implantation failure is immunotolerance failure caused by immune dysregulation [27, 28], implying that exogenous supplementation of ULF-EVs has great potential to improve implantation failure.

### MEP1B protein enriched in ULF-EVs may improve embryo implantation by promoting trophoblast cell proliferation and migration

**Fig. 6:**
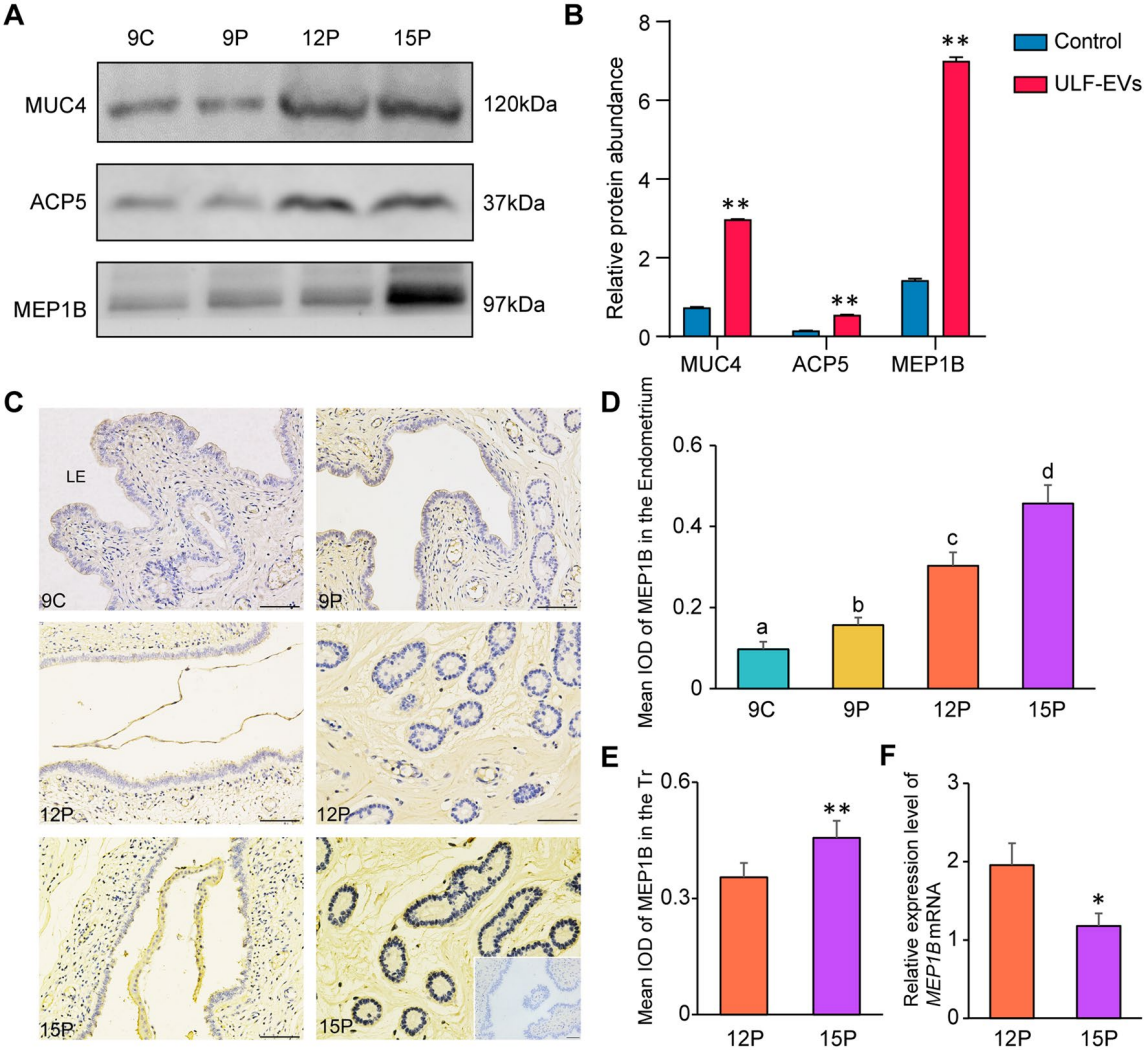
Representative proteins in ULF-EVs whose abundance increased with pregnancy development also significantly increased after ULF-EVs treatment of pTr2 cells. **A** Western blot analysis of MUC4, ACP5 and MEP1B in ULF-EVs. **B** Relative abundance of MUC4, ACP5 and MEP1B in pTr2 cells treated with ULF-EVs. **C** Images of the embryo-maternal interface stained with MEP1B antibodies. MEP1B was obviously expressed in the endometrium and trophoblast. The scale bar indicates 100 nm. **D** Quantitative analysis of MEP1B by assessing the average integrated optical density (IOD) in the endometrium. The data are displayed as mean ± SD and different lowercase letters correspond to significant differences at the *p* < 0.05 threshold. **E** Quantitative analysis of MEP1B by assessing IOD in the trophoblast. Asterisks indicate significant differences (mean ± SD) between 12 and 15P (**p < 0.01). **F** The relative expression level of *MEP1B* mRNA was determined by qRT-PCR. The data are displayed as mean ± SD and asterisk indicate significant differences (*p < 0.05)

When comparing proteins in ULF-EVs with those changed in pTr2 cells, we found that proteins in cluster 2 that increased in abundance as pregnancy progressed also increased in pTr2 cells treated with ULF-EVs (Fig. 6A, B). Of these, including MUC4, ACP5, and MEP1B, a total of 10 proteins existed in cluster 2 and proteins with significant abundance changes in pTr2 cells treated using ULF-EVs (Additional file 4: Fig. S4). Among these proteins, MEP1B with a high abundance of differential changes has been proven to be a membrane-bound metalloproteinase that cleaves various proinflammatory cytokines and extracellular matrix proteins and regulates inflammation and tissue remodeling [29, 30]. We found that the abundance of MEP1B in embryos on day 15 of pregnancy was higher than that on day 12 (Fig. 6C–F), but mRNA expression was much lower, indicating that MEP1B protein in embryos is most likely released by the endometrium, which was transferred into embryos to function through ULF-EVs.

**Fig. 7:**
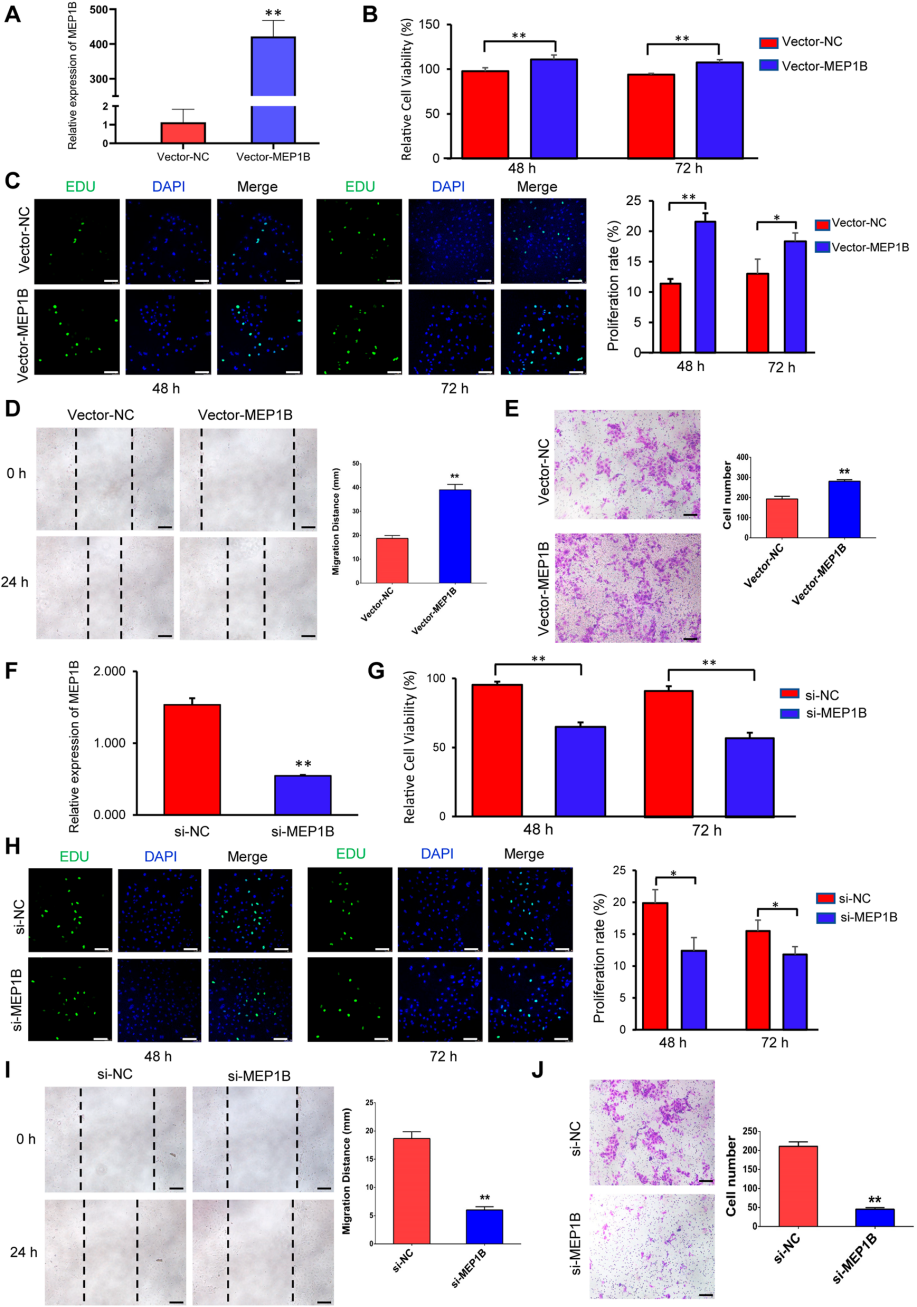
Effects of MEP1B on pTr2 cells in vitro. **A**–**E** MEP1B promotes pTr2 cell proliferation and migration. **A** The transfection efficiency of MEP1B overexpression was determined by PCR. **B** The cell viability of pTr2 cells was applied by CCK-8 assay. **C** EDU staining assay was performed to determine the cell proliferation changes after MEP1B overexpression. Scale bars = 100 µm. **D** Wound healing assay for the evaluation of migration of pTr2 cells. Scale bars = 500 µm. **E** Transwell migration assay revealed that MEP1B overexpression increased the cell numbers of migration. Scale bars = 200 µm. **F**–**J** Knockdown of MEP1B inhibits pTr2 cell proliferation and migration. **F** The transfection efficiency of MEP1B siRNA was determined by PCR. **G** The cell viability of pTr2 cells was applied by CCK-8 assay. **H** EDU staining assay was performed to determine cell proliferation changes after MEP1B knockdown. Scale bars = 100 µm. **I** Wound healing assay for the evaluation of migration of pTr2 cells. Scale bars = 500 µm. **J** Transwell migration assay indicated that knockdown MEP1B reduced the cell numbers of migration. Scale bars = 200 µm. *CCK-8* cell counting kit-8. The data were presented as mean ± SD. *p < 0.05, **p < 0.01, and Student’s *t*-test

To further investigate the biological role of MEP1B in embryos, we performed in vitro experiments. MEP1B was successfully overexpressed in pTr2 cells using plasmid vectors (Fig. 7A). We found that MEP1B dramatically boosted the proliferation rate of trophoblast cells using CCK-8 assay (Fig. 7B). EDU staining further revealed that compared to the control group, MEP1B overexpression significantly increased trophoblast proliferation (Fig. 7C). Wound healing assay migration demonstrated that overexpression of MEP1B could improve the migration distance of pTr2 cells (Fig. 7D), consistent with transwell assay results (Fig. 7E). In contrast, siRNA-mediated knockdown of MEP1B significantly decreased pTr2 cell growth and migration (Fig. 7F–J). These findings revealed that MEP1B can increase trophoblast cell proliferation and migration in vitro.

## Discussion

EVs widely exist in diverse tissue fluids, such as plasma, urine, and saliva, and participate in various physiological responses that act as a medium for information exchange between cells [7]. Indeed, there are also a large amount of EVs in the female uterine luminal. Previous studies have established that EVs generated from the oviduct and endometrium can interact with trophoblast cells, promoting their proliferation and differentiation to aid embryo development and implantation [31]. Concurrently, aberrant EV changes may cause diseases, such as endometriosis and preeclampsia, ultimately resulting in pregnancy loss [32, 33]. Owing to the unique biology and roles in cell–cell communication of EVs, recent studies have applied these nanovesicles in regenerative medicine-related treatments and as targeted delivery vehicles for drugs [34, 35]. However, the limited supply of ULF-EVs has severely hindered their clinical studies on infertility treatment, including implantation failure. In this research, pigs were used as a biomedical model for humans and ULF-EVs were successfully isolated from the uterine luminal. Subsequently, we explored the potential of these ULF-EVs as nanomedicine materials to improve embryo implantation by comprehensively characterizing the enriched proteins in ULF-EVs. Thus far, our results provide an important theoretical reference for the further use of ULF-EVs in implantation failure treatment.

We identified a total of 1549 proteins by characterizing these enriched proteins in ULF-EVs over four periods. Notably, most of these proteins arise in the cytoplasm or extracellular space, and some are present in several membranes, including the plasma membrane, endomembrane system, and organelle membrane. These proteins derived from the cytoplasm or extracellular space are most likely cargo proteins encapsulated in ULF-EVs that facilitate information exchange between cells. In contrast, those located on a series of biological membranes may assist ULF-EVs in maintaining membrane vesicle structure and medium function [36, 37]. When EVs are used as therapeutic or drug targeting vehicles, it is necessary to ensure the integrity of these membrane structural proteins to maintain the nanovesicle structure.

Furthermore, we identified two distinct groups of proteins whose abundance fluctuated gradually during pregnancy development by clustering these ULF-EVs proteins. The class of proteins whose abundance declined was considerably enriched in activities involving protein regulation, such as proteolysis or glutathione metabolism. Previous studies have shown that pericellular proteolysis plays a role in numerous aspects of ontogeny, including ovulation, fertilization, implantation, cell migration, tissue remodeling, and repair. Delicate proteolysis is required for tissue integrity and homeostasis maintenance [38, 39]. Simultaneously, recent research has demonstrated that proper protein hydrolysis can successfully promote embryo implantation [40]. Another significant class of proteins with steadily increasing abundance levels was enriched in some transport mechanisms, showing that accurate cargo exchange between the maternal and embryos is critical for successful implantation [41, 42]. Therefore, we concluded that the cargo proteins in ULF-EVs improved the embryo implantation.

To investigate the effect of ULF-EVs on embryo implantation, we treated embryonic trophoblast cells with ULF-EVs in vitro. Notably, we found that some biological processes related to immune response were visibly regulated. Indeed, the embryo, as a semi-allograft, requires an appropriate inflammatory response in utero to maintain homeostasis [43]. Abnormal inflammatory responses can cause diseases during pregnancy, leading to miscarriage [44]. In the present study, we found that supplementation with ULF-EVs significantly modulated inflammatory responses, suggesting the potential reliability of ULF-EVs as a natural medicine to improve the pregnancy environment.

**Fig. 8:**
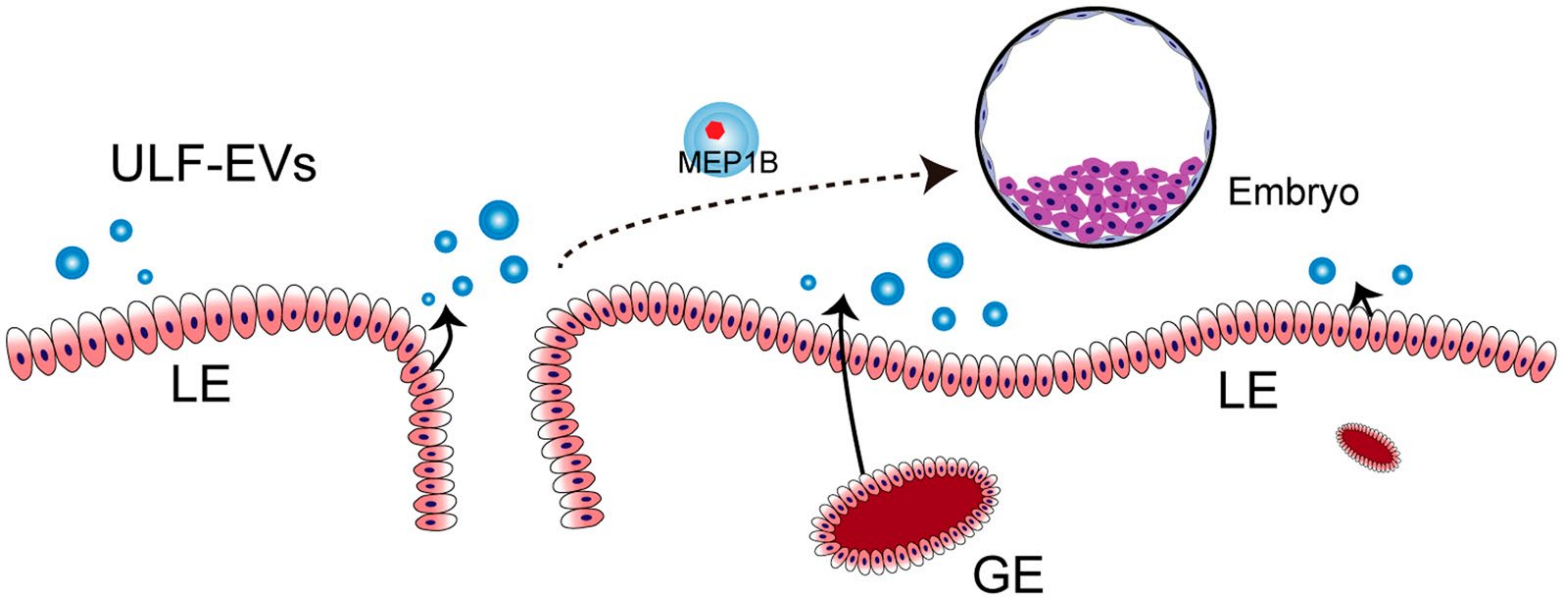
The proposed mechanism based on our results. Protein MEP1B is encapsulated by ULF-EVs, which are secreted from LE and GE into the uterine lumina and subsequently transported into the embryo

Interestingly, we found that proteins in ULF-EVs that increased in abundance with pregnancy development also increased in pTr2 cells treated with ULF-EVs, which again suggests that ULF-EVs can serve as nanomedicines for cargo encapsulation. These proteins are targeted for delivery to the embryos to improve embryo implantation. MEP1B, a cargo protein, is often expressed and plays a critical role in various epithelial cells [45–48]. Based on the protein abundance and mRNA expression level of MEP1B in the endometrium and embryos, we speculated that a part of MEP1B protein in embryos originated from the endometrium and was then transported to the embryos through ULF-EVs to perform its function. Previous studies have also demonstrated that MEP1B plays a crucial role in the communication between embryos and the endometrium and is assumed to contribute cooperatively to embryo attachment [49]. Our subsequent investigations proved that MEP1B promotes trophoblast proliferation and migration, proposing a hypothesis that MEP1B is released by LE and GE into ULF-EVs and then transferred into embryos (Fig. 8). Increased MEP1B protein levels in embryos favor embryo development, migration, and attachment and promote implantation. Unfortunately, MEP1B function was only validated in vitro in this study, and a further study should use MEP1B protein from ULF-EVs to confirm its roles.

## Conclusion

In summary, this study used a pig model to demonstrate that the uterine luminal of pigs is enriched with ULF-EVs, comprehensively characterized the proteins in ULF-EVs, and revealed their roles in embryo implantation. The feasibility of exogenous supplementation of the potential endogenous drug ULF-EVs for implantation failure treatment is then discussed. Through these experiments, we hope to provide a reference for the clinical application of ULF-EVs in treating infertility-related diseases.

## Supplementary Information

**Additional file 1: Fig. S1.** Morphology of embryo development during implantation in early pregnancy of pigs. A H&E staining of uterine sections on days 9, 12 and 15 of pregnancy. The scale bar indicates 100 nm. B The morphology of pig embryos on days 9, 12 and 15 of pregnancy. 9P, day 9 of pregnancy; 12P, day 12 of pregnancy; 15P, day 15 of pregnancy; Tr, trophoblast; LE, luminal epithelium; GE, glandular epithelium: UL, uterine luminal.

**Additional file 2: Fig. S2.** Isolation of pig ULF-EVs by OptiPrep™ density gradient ultracentrifugation.

**Additional file 3: Fig. S3.** PPI network of proteins in clusters 1 and 2. Each circle represents a protein. Blue represents the protein in cluster1 and red represents the protein in cluster2. The size of circle represents the number of connections between the protein and other proteins.

**Additional file 4: Fig. S4.** Venn diagrams of proteins with significant abundance changes in pTr2 cells treated using ULF-EVs and proteins in cluster 2.

**Additional file 5: Table S1.** The specific primers for qRT-PCR.

**Additional file 6: Table S2.** Relative abundance of proteins in the uterine luminal fluid-Evs.

**Additional file 7: Table S3.** Clustering of proteins in the uterine luminal fluid-Evs.

**Additional file 8: Table S4.** GO enrichment and KEGG pathway analysis of proteins in clusters 1 and 2.

**Additional file 9: Table S5.** Protein-protein interaction in clusters 1 and 2.

**Additional file 10: Table S6.** Differentially abundant proteins in PTR cells treated with ULF-Evs.

**Additional file 11: Table S7.** GO enrichment and KEGG pathway analysis of differentially abundant proteins in PTR cells treated with ULF-Evs.

## Data Availability

The datasets supporting the conclusions of this article are included within the article and its additional files.
