# Supplementary figures and images for: Uterine luminal-derived extracellular vesicles: potential nanomaterials to improve embryo implantation
Source: J Nanobiotechnology. 2023 Mar 7;21:79. doi: 10.1186/s12951-023-01834-1 (PMC9990359; doi:10.1186/s12951-023-01834-1)

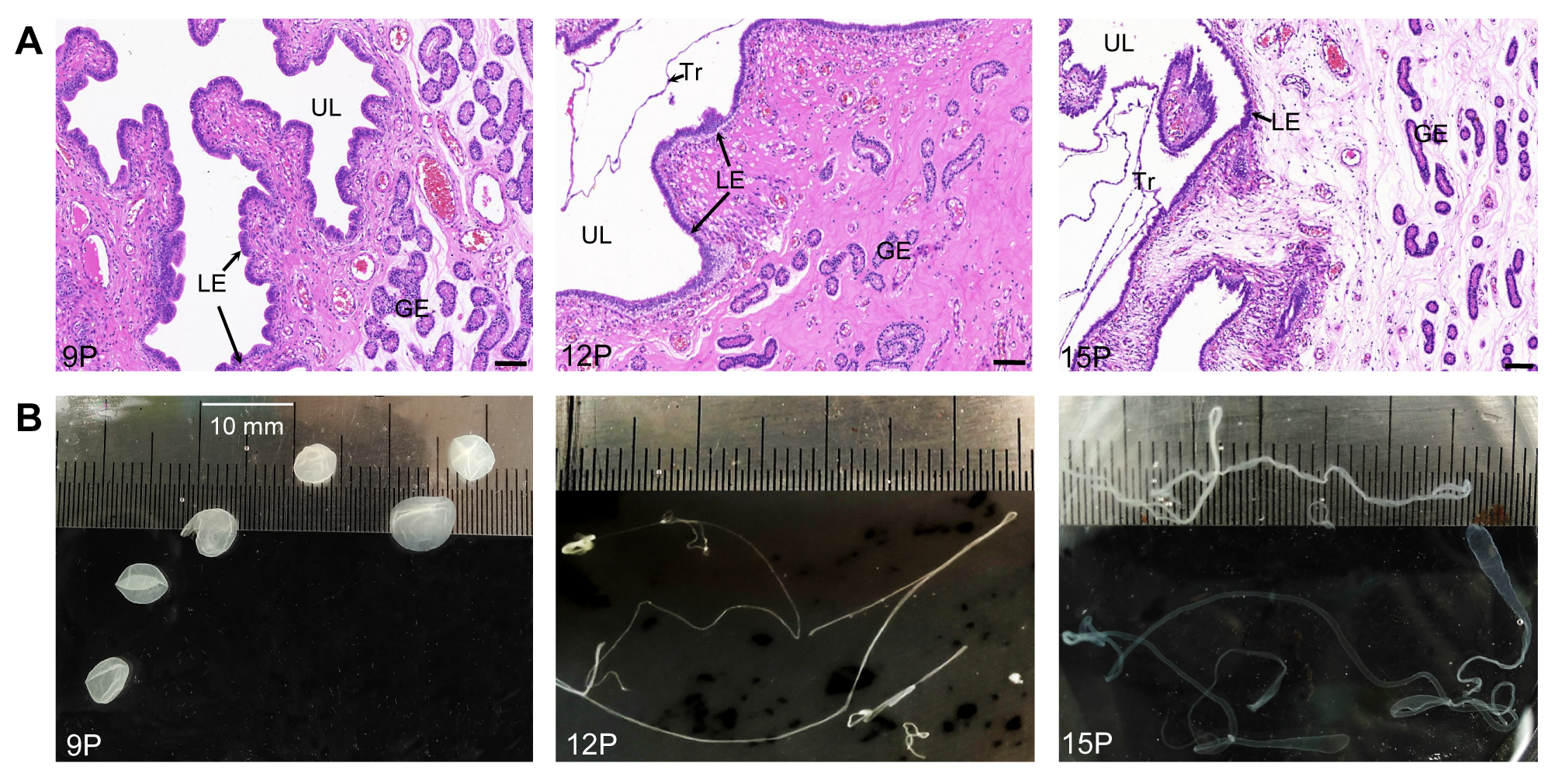

Supplement: Supplementary file 1 — Additional file 1: Fig. S1. Morphology of embryo development during implantation in early pregnancy of pigs. A H&E staining of uterine sections on days 9, 12 and 15 of pregnancy. The scale bar indicates 100 nm. B The morphology of pig embryos on days 9, 12 and 15 of pregnancy. 9P, day 9 of pregnancy; 12P, day 12 of pregnancy; 15P, day 15 of pregnancy; Tr, trophoblast; LE, luminal epithelium; GE, glandular epithelium: UL, uterine luminal. [file 12951_2023_1834_MOESM1_ESM.png]

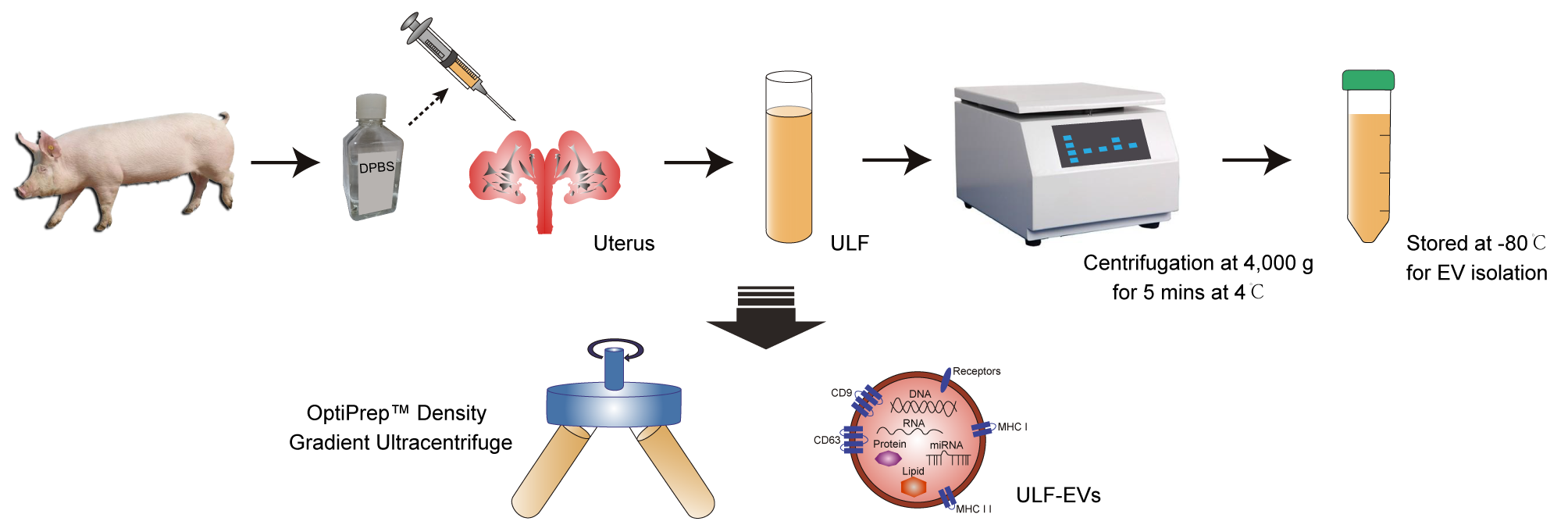

Supplement: Supplementary file 2 — Additional file 2: Fig. S2. Isolation of pig ULF-EVs by OptiPrep™ density gradient ultracentrifugation. [file 12951_2023_1834_MOESM2_ESM.png]

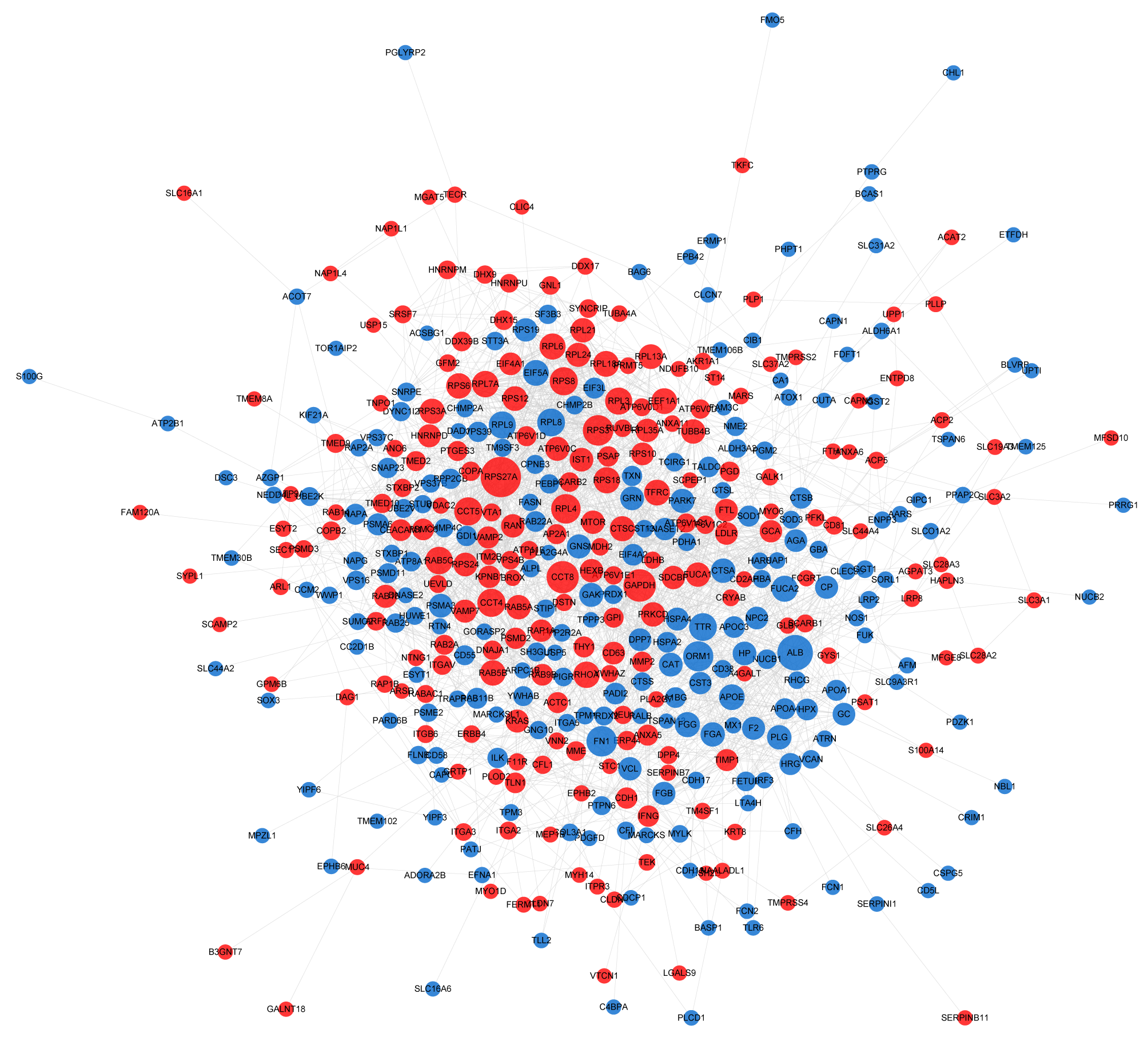

Supplement: Supplementary file 3 — Additional file 3: Fig. S3. PPI network of proteins in clusters 1 and 2. Each circle represents a protein. Blue represents the protein in cluster1 and red represents the protein in cluster2. The size of circle represents the number of connections between the protein and other proteins. [file 12951_2023_1834_MOESM3_ESM.png]

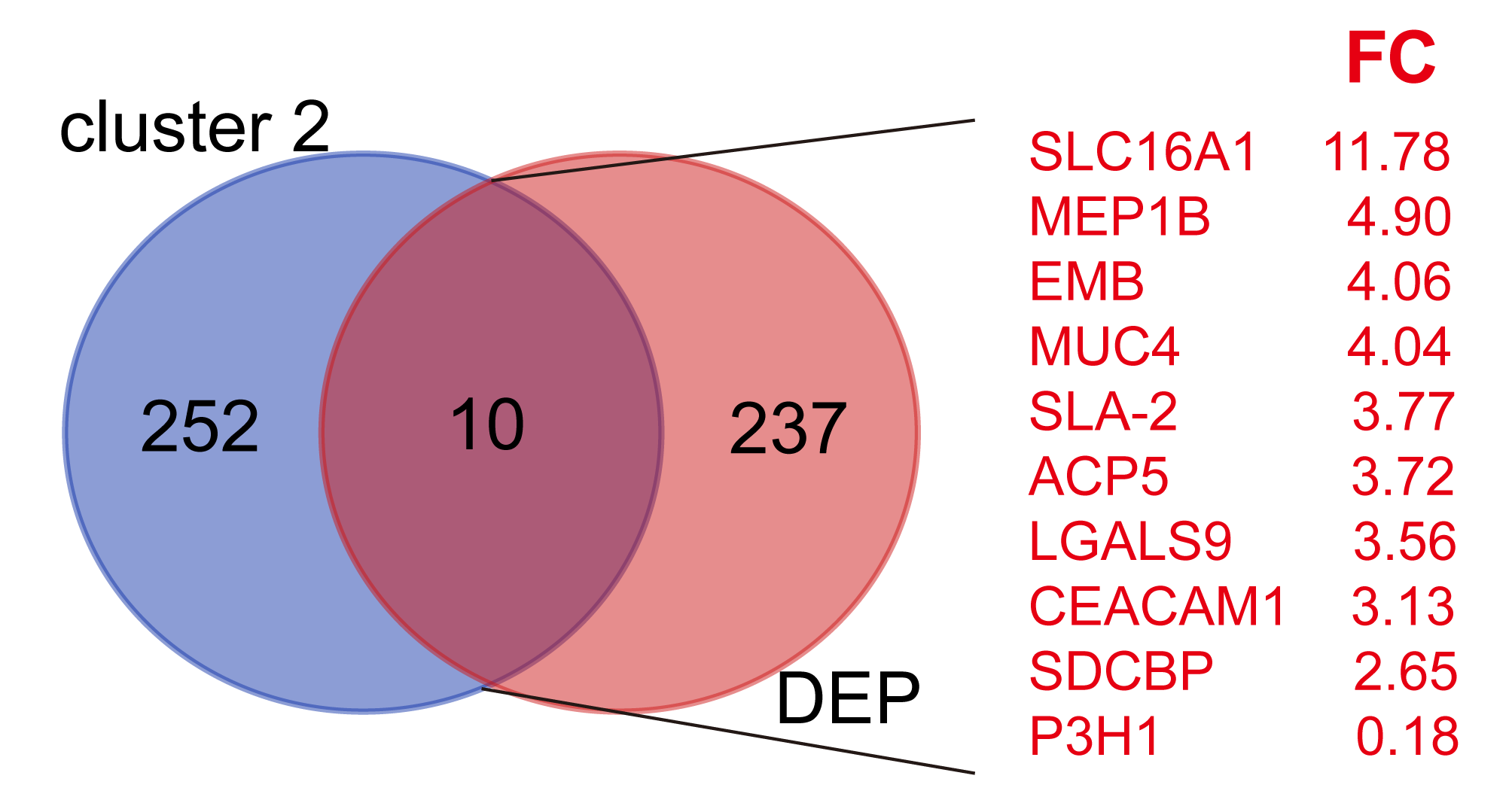

Supplement: Supplementary file 4 — Additional file 4: Fig. S4. Venn diagrams of proteins with significant abundance changes in pTr2 cells treated using ULF-EVs and proteins in cluster 2. [file 12951_2023_1834_MOESM4_ESM.png]
